# Acquired Aplastic Anemia as a Clonal Disorder of Hematopoietic Stem Cells

**DOI:** 10.1007/s12015-020-09971-y

**Published:** 2020-04-08

**Authors:** Katarzyna Brzeźniakiewicz-Janus, Joanna Rupa-Matysek, Lidia Gil

**Affiliations:** 1 Department of Hematology, Multi-Specialist Hospital Gorzów Wielkopolski, Faculty of Medicine and Health Science, University of Zielona Góra, Gorzów Wielkopolski, Poland; 2 Department of Hematology and Bone Marrow Transplantation, Poznań University of Medical Sciences, Poznań, Poland

**Keywords:** Aplastic anemia, AA, Hematopoietic stem cells, HLA-mediated immunity mutations, Defective telomerase function

## Abstract

Aplastic anemia is rare disorder presenting with bone marrow failure syndrome due to autoimmune destruction of early hematopoietic stem cells (HSCs) and stem cell progenitors. Recent advances in newer genomic sequencing and other molecular techniques have contributed to a better understanding of the pathogenesis of aplastic anemia with respect to the inflammaging, somatic mutations, cytogenetic abnormalities and defective telomerase functions of HSCs. These have been summarized in this review and may be helpful in differentiating aplastic anemia from hypocellular myelodysplastic syndrome. Furthermore, responses to immunosuppressive therapy and outcomes may be determined by molecular pathogenesis of HSCs autoimmune destruction, as well as treatment personalization in the future.

## Introduction

Acquired aplastic anemia is a rare disorder presenting with bone marrow failure syndrome due to autoimmune destruction of early hematopoietic stem cells and stem cell progenitors [1, 2]. It has been shown that acquired AA may predispose to myelodysplastic syndrome and leukemia in approximately 15–16% of cases as a consequence of clonal hematopoiesis and evolution [3, 4]. In Europe, the incidence is about two cases per million per year, while in Asia there are about three times as many cases when compared to Europe [5–7]. Cases among males and females occur equally and most cases are observed in the first three decades of life. The name of the disease, aplastic anemia (AA) is misleading because it only suggests anemia whereas pancytopenia is often presented. The term ‘bone marrow failure’ is a broader concept, and the cause may not only be AA, but hypocellular myelodysplastic syndrome (MDS) or acute myeloid leukemia (AML). Overall, AA can be divided into congenital and acquired with hematopoietic stem cells (HSC) immune-mediated destruction [8]. The inherited forms are rare and mainly include; Fanconi anemia, congenital keratosis, congenital pure red cell aplasia and Schwachman-Diamond syndrome as a result of genetic lesions leading to diminish HSC’s ability to repair DNA [9].

It was originally thought that AA was associated with damage to the haematopoiesis leading to the loss/destruction of hematopoietic stem cells (HSCs) by the following identifiable processes such as autoimmune mechanisms, direct damage to HSCs by drugs, chemicals or irradiation; virus infection and some inherited or acquired clonal and genetic disorders resulting in peripheral pancytopenia and a profound hypocellular bone marrow composed of fat cells and stroma [10]. However, autoimmune damage to HSCs is present in the majority of cases of AA, regardless of whether another underlying cause has been identified, responses to immunosuppressive therapy support this hypothesis [11–13].

Besides the primary role of cytotoxic T lymphocytes with the impact of interferon gamma and TNF on inhibition of hematopoietic stem cell colony production and enhance expression of FAS (first apoptosis signal) receptor resulting in HSC immune-mediated destruction [14–19]. There is also evidence of a deficiency and/or dysfunction of T regulatory cells, this is impacted by class I and II HLA, NK cells and autoantibodies, which are also involved in the immune destruction of HSCs in AA [20–24].

## Clonal Haematopoiesis of Hematopoietic Stem Cell

There are data on the intrinsic abnormalities of hematopoietic stem cells and progenitors in patients with AA. A decreased number of hematopoietic stem cells and their progenitors, measured as myeloid colony-forming cells, with defective stem cell function with regard to repopulating ability and increased apoptosis [2, 25, 26], and autophagy impairment in adult bone marrow CD34^+^ cells of patients with AA were observed [27]. An underlying stem cell abnormality has been postulated in patients with improved peripheral blood counts but persistent decreases in progenitor cells after treatment [28]. Genetic factors play an important role in the pathogenesis of AA with regard to uniparental disomy in 6p, somatic cell mutations, specific somatic mutation in HLA class I and class II genes, shorter telomerase with telomerase gene mutations and genetic susceptibility [8].

Some recurrent somatic alterations have been demonstrated in patients with acquired AA. One of these alterations is copy number – the neutral loss of heterozygosity of chromosome arm 6 p (6p CN-LOH), which is the major histocompatibility complex (MHC) region reach in the multiple human leucocyte antigen (HLA) loci. The 6p CN-LOH is more frequent in AA compared to other bone marrow failure syndrome [29]. The 6p CN-LOH was detected in only 13% of AA in the Japanese population [30]. Although, it may be hypothesised that this may lead to an immune escape of hematopoietic cells without certain HLA alleles, due to the presence of many other genes in this region [30, 31], a specific link between acquired 6p CN-LOH and development of AA was not established. Furthermore, as a result of the effect of T cell directed immunosuppressive therapy, HLA-class I autoimmunity was proposed. Recently, it has been identified that HLA loss is not only sufficient for clonal expansion but also does not prevent clonal evolution. Babushok et al. identified somatic mutation in HLA class I genes including 4 HLA alleles; HLA-A*33.03, HLA-A*68.01, HLA-B*14:02, and HLA-B*40:02, related to more severe disease and predisposing to other chromosomal abnormalities and clonal changes, thus supporting the link between inherited abnormalities and clonal evolution in the course of AA [32]. Also, HLA class II gene related autoimmunity has been revealed [2, 32–37]. Comparing AA and hypoplastic MDS patients, including MDS secondary to AA, using SNP array–based karyotyping, Afable et al. reported lesions involving the HLA locus suggestive of clonal immune escape in some AA patients [38]. Depending on the age of patients with acquired AA, different pathomechanisms are predominant, such as HLA mutation in children with a young onset of AA, whereas MDS-association mutation can be identified in the older population [39–42]. This may also explain the observation of a lower rate of secondary MDS among the children population with a young onset of AA. Moreover, due to identification of HLA-B*40:02 in cohorts of Asian patients [43, 44], this may explain the higher incidence of AA in East Asia, approximately 2–3 fold higher in comparison to the European population [5–7]. On the other hand, other HLA class II gene mutation has been identified in patients with AA in North America, such as HLA-B*14:02, supporting the role of HLA-mediated immunity [32]. It should be emphasised that the presence of HLA risk alleles increases the risk of a more serious course of disease, clonal evolution and complications. Conversely, in two meta-analyses the impact of the role of granulocyte colony-stimulating factor in immunosuppressive therapy of AA on clonal evolution has not been determined [45, 46]. Recently, data show that 6p- CN-LOH may confer a survival advantage to hematopoietic stem progenitor cells clones (HSPCs) with age-related somatic mutations, leading to the clonal expansion of mutant HSPCs [47].

## Telomerases Abnormalities

Telomeres are protective chromosome caps that shorten during cell replication and following exposure to stressors. One biomarker that has been used to assess the cumulative effects of these conditions experienced in early and late life is the length of telomeres [48]. Because telomere shortening leads to cell proliferative arrest and apoptosis, bone marrow failures, including AA, are associated with inherited mutations in telomere repair or protection genes [49]. Calado et al. revealed that short telomeres result in chromosomal instability leading to short telomeres producing end-to-end chromosome fusion, nonreciprocal translocations, and aneuploidy in hematopoietic cells and preceding malignant evolution in human aplastic anemia [50]. To resist the attrition, germ-like cells utilize telomerase reverse transcriptase (TERT), telomerase RNA component (TERC) telomerase genes, and the stabilizing protein dyskerin (DKC1) to assemble the telomerase complex and maintain telomere length [51]. Peripheral granulocyte and mononuclear cell telomere shortening are found in approximately 35% of AA patients indicating a defect in the HSPCs [52, 53]. Furthermore, to evaluate the telomeres and their impact on dysfunctional hematopoietic stem cells in the AA phenotype, in in vitro model mimics two typical features of the AA were built [54]. Not only did AA patients have shorter telomere length compared to the health control, but also AA patients with somatic mutations had shorter telomere lengths, compared with patients without mutations [55]. Mutations in TERT, TERC and DKC1 are associated with critical telomere length loss of HSPCs in AA patients [56, 57]. Only a small subset of AA patients harbored the same genes found in dyskeratosis congenita including TERC and TERT - associated with impair telomer length [51, 56, 57]. Other telomerase genes such as TERF1 and TERF2 have been identified in AA patients [58]. Although, heterozygous variants *RTEL1* (regulator of telomere elongation helicase 1) were identified in bone marrow failure, which included AA (1–2%), they were likely benign or of uncertain significance [59].

It has been suggested that telomere length at diagnosis is correlated with outcome [60–62]. Moreover, a few studies reported an inadequate response to immunosuppression in AA patients with shorter telomer length [53, 63, 64] or *TERT* or *TERC* mutations [56, 65] and transformation to MDS/AML [50, 61, 66, 67]. Dumitriu et al. showed that accelerated telomere attrition in the setting of a decreased hematopoietic stem and progenitor cells (HSPC) pool is characteristic of early myeloid oncogenesis, specifically for chromosome 7 loss, particularly in MDS/AML after treatment of AA, and provides a possible mechanism for the development of aneuploidy [62]. In addition to telomer abnormalities, when compared to healthy volunteers, CD34+ HSPCs from patients with AA showed down-regulation of several cell cycle “checkpoint” genes, such as Fanconi anemia complementation group (*FANCG), c-myb*, *c-myc*, cyclin-dependent kinase 6 (CDK6), CDK2 and cyclins E and A which would also be consistent with the ultimate development of premalignant or aneuploid cells and conversion to MDS/AML [68]. It has been hypothesised that these may provide additional mechanisms explaining the inability of the remaining HSPCs to replicate competently and ultimately compensate for immune-mediated destruction [69]. The identification of the AA secondary to telomerase variants/mutations is very important due to, presumably, a less likely response to immunosuppressive therapy and the patient may be a candidate to upfront HLA-matched sibling or unrelated donor allogeneic bone marrow transplantation [64, 70].

In has been shown that sex hormones or other pharmacologic agents might reduce the risk of clonal evolution inducing therapeutic upregulation of telomerase [71].

## Cytogenetics Abnormalities

Although, a number of studies have reported that cytogenetic abnormalities occur from 4 to 13% of AA [3, 72–76]. It must be emphasised that due to hypocellularity of bone marrow these results may be underestimated. Furthermore, cytogenesis abnormalities may be reported at the initial diagnoses or later developed cytogenesis abnormalities with or/without progression to MDS/AML [77]. It is known that severe AA is frequently associated with monosomy 7 (−7), occurring in up to 13% of AA cases and is associated with poorer prognosis and high risk of progression to MDS or AML [75, 78]. Dumitriu et al. reported that patients who evolved to MDS and AML showed marked progressive telomere attrition before the emergence of −7 [62]. The karyotypes most frequently encountered in MDS secondary to AA involved chromosomes 6, 7 and 8 with evolution rates of approximately 10–15% in 10 years [78]. On the contrary, trisomy 8, commonly observed in other myeloid malignancies, with an occurrence of 1.3–6.7%, is associated with a favorable response to immunosuppressive therapy and lower risk of evolution to MDS or AML [62, 75, 79]. Also, Del(13q), which occurs in 0.4–1.8% of AA cases, is consider to be associated with a favorable response to immunosuppressive therapy [80–82]. Other cytogenetic abnormalities are less commonly detected, some of these are shared with myeloid malignancies, such as del(5q), whereas others, including trisomy 6, trisomy 15 are rarely seen in AML/MDS and their clinical relevance should be established [28].

## Skewed X-Chromosome Inactivation

It has been reported that female patients with AA exhibited a clonal X-chromosome inactivation, not only a polyclonal immunoreceptor rearrangement pattern of T cell receptor gamma and an immunoglobulin heavy chain gene but also clonal somatic mutations in up to 32% of cases can be found [83–85].

## Somatic Mutations

Although AA was regarded as a non-clonal disorder of hematopoietic stem cells and stem cell progenitors, recently several studies reported the high frequency of clonal abnormalities and haematopoiesis [55, 76, 85–87]. In general, clonal haematopoiesis is defined by a disproportionately large fraction of hematopoietic cells arising from a single stem cell or a multi-potent hematopoietic progenitor and can be detected by identifying genetic changes (e.g., mutations or chromosomal abnormalities) developed during human life known as clonal evolution. Recent advances in genome analysis, including single nucleotide polymorphism (SNP-As) arrays, targeted new generation sequencing (NGS), whole exom-sequencing (WES) and NGS-based HLA typing, have revealed somatic mutations in patients with AA which confirm the association of clonal haematopoiesis in the pathogenesis of AA [88].

In most cases, the clinical relevance of somatic mutations in AA has been studied in the context of prognosis, response to the immunosuppressive therapy and differences between severe and non-severe AA and the risk of progression to MDS/AML. Kulasekararaj et al. postulated that somatic mutations are related to the transformation to MDS/AML which has been found in 40% of cases and also the longer duration of the disease in comparison to the group without somatic mutations [55]. Yoshizato et al. performed next-generation sequencing and array-based karyotyping using 668 blood samples obtained from 439 patients with aplastic anemia, the tests reported clonal hematopoiesis of stem cell with somatic mutations in 47% of patients, mainly acquired mutations [89]. On the other hand, depending on the study population age, other studies did not confirm the same high incidence of somatic mutations among AA patients [76, 85], particularly in the pediatric population [85]. Negoro et al. reported that although some mutations are typical for MDS, they may reflect clonal hematopoiesis and may have potential to evolve from AA to secondary MDS but not in all cases. Some these mutations ale likely to predict secondary MDS [90].

Overall, clonal haematopoiesis of stem cell in the course of AA can be divided into two groups [76, 89, 91]. The first group includes patients with recurrently mutated genes such as clonal haematopoiesis of indeterminate potential (CHIP), including DNMT3A and ASXL1 [75, 89, 92, 93]. This group of mutations has been found to be at risk of inferior prognosis with a higher risk of evolution to MDS and AML [55, 89]. DNMT3A mutation is involved in DNA methylation [94, 95], ASXL1 belongs to the polycomb family influencing chromatin structure and function [94, 96].

The second group of patients is younger and it is characterised by the most commonly mutated genes in AA include PIGA, BCOR, BCORL1, which are known to be relatively benign/indolent mutations [89]. The PIGA gene plays a role in biosynthesis of glycosyl-phosphatidyl-inositol (GPI), the mutation inactivates its functions [94, 97]. BCOR is a co-repressor of BCL6, involved in B cell development, and has a mostly inactivating function in AA patients [94, 98].

In general, in other clonal haematopoiesis these mutations are not found and are known to be unique to AA. Probably, they may promote the autoimmune response of the T cells. The second group of mutations is of great importance for clinicians because PIGA and BCOR may predict a favourable response to immunosuppressive therapy with improved progression-free survival and overall survival. Other mutations associated with a poor response to immunosuppressive therapy include: DNMT3A, ASXL1, JAK3, RUNX1, TP53, CSMD1 [99].

Although some mutations may drive clonal expression in other disorders such as TET and JAK2, the impact on clonal evolution in patients with AA is less prominent.

Due to analyses of the somatic mutations primarily involved in the pathways of immunity and transcriptional regulations, Babushok et al. reported somatic mutations in 72% of all analyzed AA patients including 66% of pediatric AA, while MDS-associated genes were only in 9% [86]. One of the most frequently reported somatic mutations, found in nearly half of AA patients, is PIGA, which results in clonal populations of cells lacking cell surface proteins linked to a glycosylphosphatidylinositol (GPI) anchor due to somatic loss-of-function mutations, one of a number of drivers of clonal evolution in AA and paroxysmal nocturnal hemoglobinuria (PNH) [100–102]. In 25% of AA patients, progression into clinical hemolysis after immunosuppressive therapy is observed after clone size raises up to 37% for erythrocytes and 28% for granulocytes [101]. Babushok et al. identified two potential driver mutations in HLA alleles, STAT5B and CAMK2G, associated with translational consequences in pathways of hematopoietic growth and immunity [29].

The differences between patients with severe and non-severe AA in genomic features were recently reported by Patel J et al [4]. Although at least one mutation was identified in 19% of patients with AA at the time of diagnosis, independently of the severity of the AA, these mutations included DNMT3A, PIGA, SRSF2 and CEBPA and there were no differences in the average number of mutations. Patients with AA had a higher mutation rate/more pronounced mutational burden in comparison to moderate AA (56% vs 19%) which corresponds to the unstable hematopoietic clones and higher risk of evolution [4].

Imi et al. reported that in AA patients, hematopoietic stem progenitor cells (HSPCs) clones lacking an HLA class I allele which escape the cytotoxic T lymphocyte (CTL) attack are essentially free from somatic mutations related to myeloid malignancies and are able to support long-term clonal hematopoiesis without developing driver mutations in AA patients unless HLA loss occurs in HSPCs with somatic mutations [47].

## The Role of Inflammaging in Pathogenesis of AA

The process of “inflammaging” refers to chronic, low-grade, sterile inflammation that develops in hematopoietic tissues with advanced age [103]. This leads in the patients to the increased activity of innate immunity and a decrease in acquired immunity. Inflammaging is a consequence of cellular turnover and chronic cellular stress in the absence of infection and is driven by pro-inflammatory mediators including TNF-α and IL-6 that are part of the senescence-associated secretory phenotype (SASP) [104]. In addition in pathogenesis of inflammaging are involved several danger associated molecular pattern molecules (DAMPs) including extracellular ATP, oxidatively modified DNA; and aggregated proteins released from damaged cells [105]. All these DAMPs lead to activation of the Nlrp3 inflammasome, that drives process of inflammaging in the all tissues including bone marrow [106–108]. Activation of Nlrp3 inflammasome leads to release from innate immunity cells two pro-inflammatory interleukins IL-1β and IL-1 [105–108]. While as demonstrated IL-1β signaling decreases erythropoietin secretion in the kidney [109], IL-18 induces interferon gamma (IFN-γ) expression, which along with IL-1α synergistically inhibits erythroid colony formation [110]. This enhanced basal level of inflammaging may lead to an increased risk of clonal hematopoiesis and as a consequence spontaneous anemia anemia what will be discussed in the next paragraph.

## Age-Related Clonal Hematopoeisis of Stem Cells Predisposing to AA

The increased incidence of AA in the elderly may be associated with age-related alterations in hematopoietic stem cells and progenitor cells (HSPCS) [40] including X-chromosome inactivation in ageing females. Age-related clonal haematopoiesis (ARCH) is reported not only in elderly patients with unexplained cytopenia, but also in survivors of AA, it has an increased risk of evolving to MDS or AML [89]. The next-generation sequencing and array-based karyotyping obtained from 439 patients with aplastic anemia showed clonal hematopoiesis of stem cell with somatic mutations in 47% of patients with AA, mainly acquired mutations [89].The incidence of variant allele frequency in some genes has been reported to be up to 31% for ASXL1. This may suggest the positive clonal selection of bone marrow hematopoietic cells with ARCH clones and also contribute to the premalignant state in these individuals.

## Circulating Exosomal microRNAs

Inflammaging is also characterized by release of microvesicles and exosomes from stressed or damaged cells [111]. These small circular membrane fragments that contain cell cytoplasma-derived mRNA, microRNA and proteins may be of diagnostic value [112, 113]. Recently, a study was performed by Guidice et al. into the utility of 25 differentially expressed exosomal microRNAs, related to intracellular functions, such as metabolism, cell survival, and proliferation in differential diagnosis between patients with AA and MDS. Moreover, one microRNA, mir-126-5p, was negatively correlated with a response to therapy for aplastic anemia and patients with a higher relative expression of miR-126-5p at diagnosis had the shortest progression-free survival compared to those with lower or normal levels [114].

Another study reports a significant negative correlation between the expression of miR-144-3p and ten-eleven translocation 2 (TET2) which cause reduced osteogenic capacity of bone marrow mesenchymal stem cells taken from patients with AA, this may be a therapeutic strategy in future [115].

## Conclusion

**Fig. 1:**
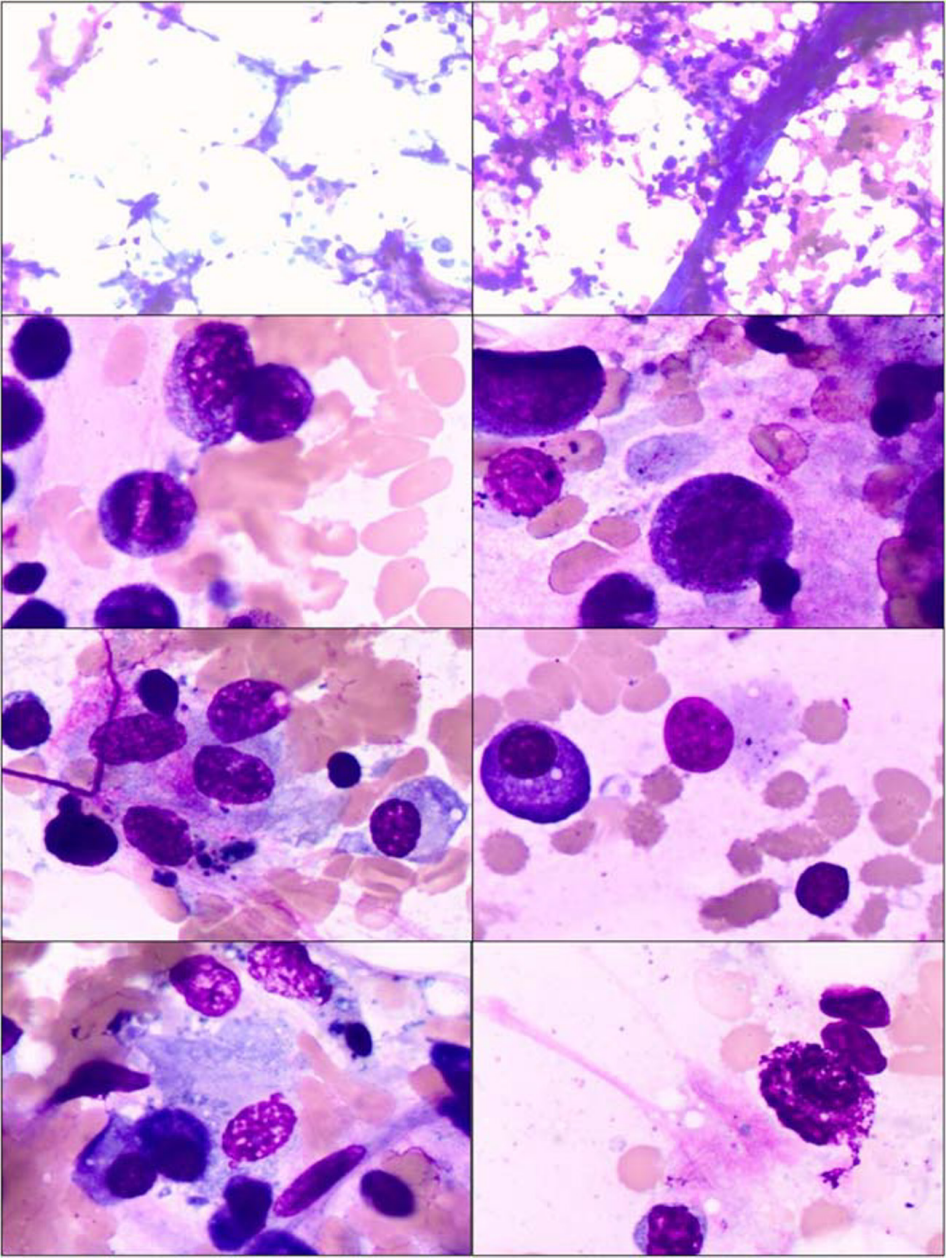
The bone marrow image shows a reduction in the number of hematopoetic cells and increase in the area occupied by adipose tissue. Bone marrow is hypocellular with a cluster of lymphoid cells. The presence of naked cell nuclei, and fragments of connective tissue, mast cells, reticulum cells and plasmocytes. Increased percentage of indolent granulocyte form (presence of granulocytes with toxic granules)

**Fig. 2:**
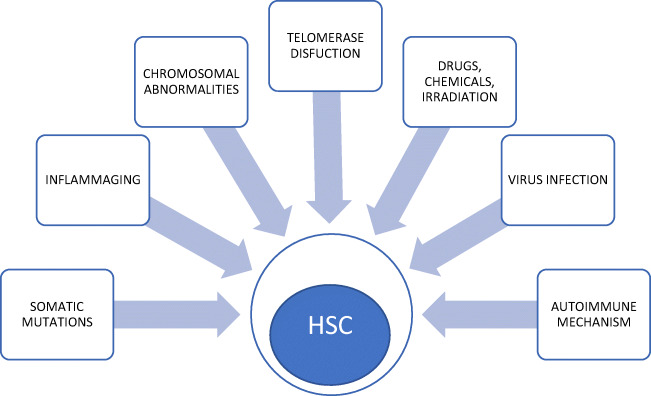
Stem cell damage factors

There are several factors contributing to the destruction of hematopoietic stem cells in the pathogenesis of AA. Recent advances in understanding the molecular pathogenesis of AA, which tends to be a clonal disorder of hematopoietic stem cells and their progenitors with somatic cell mutations, including HLA-mediated immunity mutations, defective telomerase function, inherited and acquired cytogenetic aberrations, may allow, in some situations, for the prediction of the results of immunosuppressive therapy and a forecasting of an unfavourable outcome. Identification of somatic mutation predisposing to MDS/AML or accelerated telomere attrition preceded aneuploidy and malignant transformation in the course of AA may allow for the appropriate and well-timed management of patients at high risk of clonal evolution including stem cell transplantation. Figure 1 shows the bone marrow image of aplastic anemia. The stem cell damage factors are presented on Fig. 2.
